# Eotaxins and Their Receptor as Biomarkers of Colorectal Cancer

**DOI:** 10.3390/jcm10122675

**Published:** 2021-06-17

**Authors:** Monika Zajkowska, Agnieszka Kulczyńska-Przybik, Maciej Dulewicz, Kamil Safiejko, Marcin Juchimiuk, Marzena Konopko, Leszek Kozłowski, Barbara Mroczko

**Affiliations:** 1 Department of Neurodegeneration Diagnostics, Medical University of Bialystok, 15-269 Bialystok, Poland; agnieszka.kulczynska-przybik@umb.edu.pl (A.K.-P.); maciej.dulewicz@umb.edu.pl (M.D.); mroczko@umb.edu.pl (B.M.); 2 Maria Sklodowska-Curie Oncology Center, Department of Oncological Surgery with Specialized Cancer Treatment Units, 15-027 Bialystok, Poland; kamil.safiejko@gmail.com (K.S.); jumedica.onkologia@gmail.com (M.J.); marzeniedoc@yahoo.com (M.K.); leszek@kozlowski.pl (L.K.); 3 Department of Biochemical Diagnostics, Medical University of Bialystok, 15-269 Bialystok, Poland

**Keywords:** CCL11, CCL24, CCL26, CCR3, CRC

## Abstract

Colorectal cancer (CRC) is one of the most common malignancies. Despite the availability of diagnostic tests, an increasing number of new cases is observed. That is why it is very important to search new markers that would show high diagnostic utility. Therefore, we made an attempt to assess the usefulness of eotaxins, as there are few studies that investigate their significance, in patients with CRC. The study included 80 subjects (CRC patients and healthy volunteers). Serum concentrations of all eotaxins were measured using a multiplexing method (Luminex), while CCR3 was measured by immunoenzymatic assay (ELISA). CRP levels were determined by immunoturbidimetry and classical tumor marker levels (CEA and CA 19-9) and were measured using chemiluminescent microparticle immunoassay (CMIA). The highest usefulness among the proteins tested showed CCR3. Its concentrations were significantly higher in the CRC group than in healthy controls. The diagnostic sensitivity, specificity, positive and negative predictive value, and the area under the ROC curve (AUC) of CCR3 were higher than those of CA 19-9. The maximum values for sensitivity, negative predictive value, and AUC were obtained for a combination of CCR3 and CRP. Our findings suggest the potential usefulness of CCR3 in the diagnosis of CRC, especially in combination with CRP or CEA.

## 1. Introduction

Colorectal cancer (CRC) is one of the most common malignancies not only in Europe, but also around the world. According to the World Health Organization (WHO), in 2020, the global incidence of all cancers was 19.3 million new cases, with approximately 9.9 million deaths. CRC comprises about 10% and 9.4% of all cases, respectively. Unfortunately, the incidence of this cancer is slowly but steadily increasing [1]. Currently, as a part of preventive diagnostics of colorectal cancer, tests such as FOBT (fecal occult blood test), FIT (fecal immunochemical test), colonoscopy, sigmoidoscopy, computed tomographic (CT) colonography, or multi-target stool deoxyribonucleic acid (mt-sDNA) test are used [2]. Also, markers such as CEA (carcinoembryonic antigen) or CA 19-9 (carbohydrate antigen 19-9) are routinely determined in patients with CRC. However, their diagnostic sensitivity and specificity are not satisfactory. That is why establishing new, more accurate markers for CRC detection at its earliest stage is vital [3].

Eotaxins belong to a group of small proteins called chemokines. They were discovered relatively recently—less than 30 years ago in London by Williams et al. [4,5]. Therefore, the number of papers on these parameters is still insufficient to fully understand their potential usefulness in the diagnosis or monitoring of certain diseases. The most widespread and so far the best-known usefulness of these proteins has been found in the course of allergic diseases. It is closely related to their influence on cells such as eosinophils and basophils, which are the most important cells in the development and course of those diseases. All eotaxins have the ability to bind to CCR3 (C-C chemokine receptor type 3) [6,7].

However, there are only a few studies that would indicate the usefulness of these parameters in the diagnosis, monitoring, or staging of neoplastic diseases, such as colorectal cancer. Extending the availability of information on this topic is quite important due to the discrepancy of the available data. Some of the studies show a decrease in the concentration of CCL11, while others, on the contrary, report an increase in the concentration of this parameter in the course of CRC. On the other hand, for the other parameters (CCL24, CCL26, and CCR3), no work is available on their concentration in patients with CRC. However, there are works that describe the increased expression of eotaxins in neoplastic tissues. Some authors believe that those parameters increase the proliferation of neoplastic cells and their migration, and increases the expression of the receptor for eotaxins (CCR3). Activation of this receptor on endothelial cells leads to increased angiogenesis and tumor development. On the other hand, increased tissue expression of eotaxins can also lead to the recruitment of eosinophils (cells with anti-tumor activity) into the tumor environment [8]. Therefore, we made an attempt to clarify and assess the usefulness of eotaxins in patients with colorectal cancer compared to the healthy control. We have investigated serum levels, diagnostic utility (sensitivity, specificity, predictive values of positive and negative test results), and power (ROC curve analysis) of all eotaxins (CCL11, CCL24, CCL26), their receptor (CCR3), comparative tumor markers (CA 19-9, CEA), and inflammatory parameter such as C-reactive protein (CRP) in colorectal cancer detection. The data obtained in this study may prove the usefulness of the analyzed parameters in the detection of CRC.

## 2. Results

Table 1 shows the serum levels of CCL11, CCL24, CCL26, CCR3, CA 19-9, CEA, and CRP in patients with colorectal cancer and in the control group. Concentrations of CCR3, CEA, and CRP in the total cancer group were statistically significantly higher when compared to the control group (in all cases *p* < 0.05).

In order to carry out a more detailed analysis of the obtained results, we divided the study group into patients with colon cancer and rectal cancer. Performed statistical analysis with use of the Mann–Whitney U test showed similar results. In the case of the results of colon cancer vs. control group patients, statistical significance was demonstrated for CEA and CRP (*p* < 0.001 in both cases) (Supplementary Table S1). For rectal cancer, statistical significance was demonstrated for CEA, CRP, and CCR3 (*p* = 0.003; *p* < 0.001; *p* = 0.012, respectively) (Supplementary Table S2). We did not perform a statistical analysis for sigmoid cancer patients due to an insufficient number of patients in this subgroup. We also compared the results between the two subgroups (colon cancer vs. rectal cancer), but there were no statistical differences (Supplementary Table S3). Additionally, we performed a more detailed analysis with division into advancement groups. We divided the colorectal cancer group of patients into Early (TNM stages 0+I+II) and Advanced (TNM stages III+IV) CRC. Statistical analysis with use of Mann–Whitney U test in this case also revealed similar results. In analysis of Early CRC vs. control group, statistical significance was observed in the case of CEA and CRP (*p* = 0.006, *p* < 0.001, respectively) (Supplementary Table S4). In analysis of Advanced CRC vs. control group, statistical significance was observed in the case of CCR3, CEA, and CRP (*p* = 0.024, *p* = 0.002, *p* = 0.001, respectively) (Supplementary Table S5). We also tried to compare results between two TNM subgroups (Early vs. Advanced CRC), but none of the results were significant (Supplementary Table S6). Differences in the statistical significance of CCR3 in the case of rectal cancer and its absence in the case of colon cancer may be related to the differences in the biology of these two neoplasms. However, due to the insufficient number of patients with colon cancer, further analysis was carried out on the entire study group of CRC patients. The differences obtained between the two histological types should be repeated in a larger study group, which could significantly influence the development of knowledge about eotaxins, and we assume that at this point the obtained results should be treated as a pilot, preliminary study.

Table 2 shows the sensitivity (SE), specificity (SP), positive predictive value (PPV), and negative predictive value (NPV) of all tested parameters. We indicated that the highest SE from all tested parameters revealed CCR3 (68%) and this value is comparable to SE of C-reactive protein (72%) and higher than SE of commonly used tumor marker CA 19-9 (40%). Only CEA showed higher SE (80%). In the case of SP, the highest value was observed for CCL26 (92.31%). SP for CCR3 (62.07%) was comparable to results of both tumor markers (CA 19-9 and CEA) but lower than SP observed for CRP (79.31%). Positive predictive value was highest in the case of CCL24 and CCR3 (72.97% and 75.56%, respectively). These values were slightly lower than the PPV of CEA and CRP. Interestingly, almost all tested parameters (CCL11, CCL24 and their receptor) showed higher PPV than CA 19-9. The NPV was highest for CCR3 (52.94%), but slightly lower than the NPV of CEA and CRP. What is more, similar to PPV, the NPV of all tested parameters was similar or even higher than the NPV of commonly used tumor marker—CA 19-9.

According to promising results obtained for CCR3, we decided to check whether the simultaneous analysis of two parameters would significantly change the value of the diagnostic criteria. Therefore, we created two panels consisting of CCR3 with a comparative marker—CEA and CCR3 with CRP. Combined analysis of CCR3 with both parameters resulted in an increase of SE (92%; 94%, respectively) and NPV (76.47%; 82.35%, respectively) in both cases. The most favorable combination proved to be CCR3 + CRP, what may indicate the significance of the inflammatory component in the course of this malignancy.

**Figure 1:**
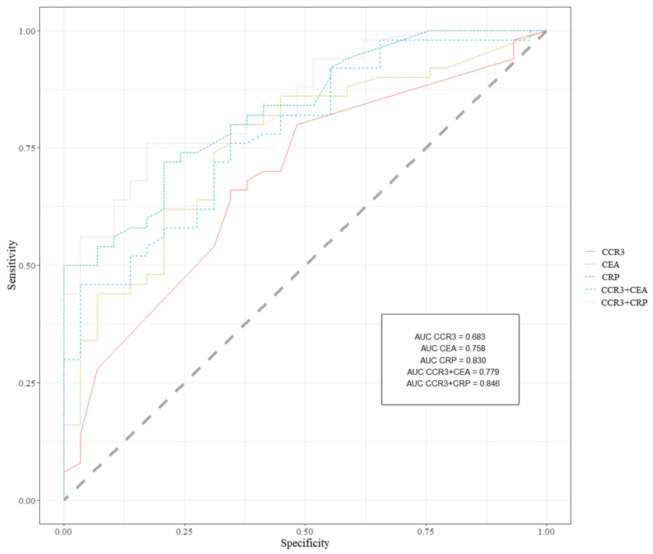
Receiver operating characteristics for all significant ROC analysis results.

The ROC curve is an illustrated relationship between the diagnostic SE and SP. The area under the ROC curve (AUC) indicates the clinical usefulness of a tumor marker and its diagnostic power. All data related to AUC has been shown in Table 3. We noticed that the CCR3 area under the ROC curve (0.683) in the total group of colorectal cancer was the highest from all the tested parameters but lower than the AUC for CEA and CRP. Moreover, similarly to previously mentioned statistical parameters, in the case of all eotaxins, the AUC was similar or even higher than the AUC of CA 19-9. Likewise, in the case of diagnostic criteria, the AUC for the simultaneous analysis of CCR3 with CEA or CRP showed a marked increase in the area under the ROC curve value (0.779; 0.846, respectively). Graphical versions of all significant ROC analysis results are shown in Figure 1. The AUCs for the tested parameters, similar to commonly used tumor markers and combined analysis, were statistically significantly larger in comparison to AUC = 0.5 (borderline of the diagnostic usefulness of the test) (*p* < 0.05 in all cases).

## 3. Discussion

Currently, little is known about the concentration and diagnostic usefulness of eotaxins and their receptor in the course of CRC. Available literature describes results that are fragmentary, characterized by a high discrepancy, or, in some cases, not entirely clear. Therefore, we decided to conduct a confirmation study on whether the examined parameters may be useful in the detection, screening, or prognosis and monitoring of the applied treatment.

Eotaxins are closely related to cells such as eosinophils and basophils. These cells are mainly involved in allergic reactions, which can be included in the group of inflammatory reactions. Neoplastic changes also begin as a result of inflammatory reactions leading to the formation of cancer. Therefore, it can be assumed that similar cytokines may appear and change their concentration as a result of both of these phenomena (allergic reactions and onset of neoplastic changes). The first references to the relationship between eosinophils and neoplastic changes were described over 100 years ago, but so far, their role in the carcinogenesis [7,8,9] has not been precisely explained. There are suspicions that the involvement of eotaxins in CRC may be related to the large number of circulating eosinophils that appear and accumulate in neoplastic tissues. TATE (tumor-associated tissue eosinophilia) has also been connected with improved prognosis in CRC and some other types of cancer such as esophageal, oral squamous cell, bladder, and prostate cancer [10,11,12,13,14,15,16]. Moreover, there are some studies that clearly indicate that tissue eosinophilia in the course of neoplastic changes may be closely related to the factors secreted directly by tumor cells. These factors can certainly include eotaxins. That is why it is so important to extend the research carried out so far on these parameters not only in the course of CRC but also in other cancers [17,18].

We indicated that serum concentrations of CCL11 in the tested group (CRC) were lower than in the control group. Despite the lack of statistical significance, similar results were obtained in the course of other experiments carried out by Wagsater et al. [19]. These researchers obtained significantly lower concentrations of CCL11 in CRC patients (which can be connected with a larger test group). In addition, they also performed IHC (immunohistochemistry) staining and examined the concentration of CCL11 in tissue homogenates. This revealed that CCL11 may accumulate in tissues, which is why its concentration can be lowered in the serum of CRC patients. By contrast, Mir et al. [20], Yamaguchi et al. [21], and Komura et al. [22] reported that CCL11 levels are higher in CRC and inflammatory bowel disease patients than in control groups. This discrepancy might be correlated with some differences between the composition of study groups, i.e., receiving corticosteroids in the case of the Mir et al. [20] study and different ethnicity [21] or smaller study group in the case of the last paper [22].

The concentration of CCL24 (Eotaxin-2) in the CRC group was higher than in the control group, but the results were not significant. It may also be connected with the small size of the tested group. Unfortunately, there were no papers that could confirm or contradict the obtained results. In the case of tissue expression of CCL24, the results of available papers were similar to those concerning CCL11. Cheadle et al. [23] revealed that in biopsy samples of CRC and samples of adjacent liver metastases (with CRC origin), the levels of CCL24 were elevated.

In the case of CCL26 (Eotaxin-3), the medians did not differ between the groups which may indicate that this parameter is not related to the ongoing neoplastic changes. However, some authors [24] indicated that, similar to CCL11 and CCL24, tissue expression of Eotaxin-3 was higher in CRC patients. What is more, its expression increased among those with TNM stage and strongly correlated with lymph node metastasis. Perhaps the small number of patients with distant metastasis (stage IV of TNM classification) in our tested group was too low to affect the entire CRC group and demonstrate statistical significance. It can be associated with a generally small number of patients at this stage. In our department, screening diagnostics for CRC are well developed. In addition, the available research methods are very sensitive, which is why cancer detection occurs at a relatively early stage. Even if a patient arrives already in stage IV, when distant metastases are detected, not every patient can be included in the study group (high BMI, no consent to research, palliative treatment). Therefore, we believe that further studies performed on a larger study group (additional patients with distant metastasis) are vital, and research on the concentrations of this eotaxin should not be discontinued.

The most interesting results were obtained for CCR3, which showed a significantly higher concentration in the serum of CRC patients when compared to the control group (*p* = 0.012). Unfortunately, we have not found any studies that would concern the concentrations of this parameter in the course of CRC. However, there are several studies that clearly indicate the usefulness of this parameter. Lan et al. [24], Cheadle et al. [23], and Cho et al. [25] unequivocally showed that the tissue expression of CCR3 in the course of this tumor is much higher when compared to healthy tissues. Interestingly, Devaud et al. [26] demonstrated that CCR3 has an anti-tumor effect correlated with the delayed growth of tumor cells. This information may be very important when we compare this study with the study by Steegenga et al. [27] showing that the tissue expression of this receptor was higher in female than in male mice. This could explain the differences in morbidity and mortality between the sexes for this type of cancer. That is why, further studies on CCR3 concentration and tissue expression should be performed.

We also measured the commonly used tumor markers (CEA, CA 19-9) and C-reactive protein concentrations in CRC patients and in the control group. CEA and CRP revealed significantly higher concentrations in the tested group when compared to healthy subjects. In the case of CA 19-9, we did not observe any significance. This is in accordance with different results obtained in the course of other experiments concerning CRC [28,29].

According to our knowledge, the present study is the first that assesses the diagnostic significance of serum CCL11, CCL24, CCL26, and CCR3 in CRC patients. We have found only one paper concerning diagnostic SE, SP (without PPV and NPV), and AUC for CCL11. In this paper, Yamaguchi et al. [20] reported that diagnostic sensitivity for CCL11 was 75.80%, specificity 66.70%, and AUC 0.714. These values were higher than ours, but the discrepancy might be connected with the size of the tested groups and their ethnicity (Japanese patients). Almost all tested parameters (CCL11, CCL24, and CCR3) showed higher SE than commonly used tumor marker CA 19-9. In the case of CEA and CRP, none of the newly tested parameters showed higher SE. In the case of SP, CCL26 showed the highest value. From the rest of tested parameters, CCR3 and CCL24 showed similar values to CEA and CA 19-9, but lower than the value for CRP. The PPV similar to SE, was higher in the case of almost all tested parameters than the PPV of CA 19-9, but lower than the PPV of CEA and CRP. The NPV, similar to SP, was highest for CCR3 but slightly lower than the NPV of CEA and CRP. What is more, similar to PPV, the NPV of all tested parameters was similar or even higher than the NPV of CA 19-9. In addition, simultaneous analysis of CCR3 + CEA and CCR3 + CRP revealed a marked increase in diagnostic SE and NPV. We noticed that the CCR3 area under the ROC curve in CRC was highest from all tested parameters but lower than AUC for CEA and CRP. Moreover, similar to previously mentioned statistical parameters, in the case of all tested proteins, AUC was comparable or even higher than AUC for CA 19-9. Similar to the diagnostic criteria (SE, SP, PPV, NPV), we observed a higher AUC value for simultaneous analysis of CCR3 with CEA and CRP. Due to the absence of appropriate data, it is impossible to discuss the above-described results.

The observed diagnostic usefulness of the parameters tested indicates that CCR3 could potentially prove to be the best of all tested proteins, especially with the combined analysis with CEA or CRP as a diagnostic panel. As CCR3 is present on the surface of endothelial cells and eosinophils, a significant increase in its concentration and tissue expression may contribute to both the intensification of angiogenesis and the influx of eosinophils, which can lead to tissue eosinophilia (important in the development of neoplastic changes). Considering the current results, it would be necessary in the future not only to expand the study group to a larger number of patients, but also to compare the obtained results with the number of circulating eosinophils, their expression in tissues, and the concentration of other parameters involved in the angiogenesis process, i.e., VEGF (vascular-endothelial growth factor).

## 4. Materials and Methods

### 4.1. Patients

The study included 50 colorectal cancer patients (CRC) diagnosed by the oncology group (Table 4). The patients were treated in the Department of Oncological Surgery with Specialized Cancer Treatment Units, Maria Sklodowska-Curie Oncology Center, Bialystok, Poland. Tumor classification and staging were conducted in accordance with the International Union Against Cancer Tumor-Node-Metastasis (UICC-TNM) classification. Colorectal cancer histopathology was based on the microscopic examination of tissue samples. Moreover, all patients were grouped according to not only tumor stage (TNM), but also depth of tumor invasion (T factor), the presence of lymph node (N factor), and distant metastases (M factor) as well as the histological grade (G factor) of the tumor. The pretreatment staging procedures included physical and blood examinations, computed tomography (CT), and, in case of patients with rectal cancer, magnetic resonance imaging (MRI) of the small pelvis. Additionally, all patients were assessed according to the Eastern Cooperative Oncology Group (ECOG) score. The blood was collected the day before the treatment (surgery, radio, or chemotherapy). The control group included 30 healthy volunteers. For each of the patients qualified for the control group, the following exclusion criteria were applied: active infections and symptoms of an infection (both bacterial and viral), other comorbidities that can affect cytokine concentrations (respiratory diseases, digestive tract diseases) or systemic diseases such as lupus, rheumatoid arthritis, or collagenosis. In addition, none of the patients included in the control group abused alcohol, smoked, or had a personal or familial history of cancer. None of the patients included (both in the study and control group) had a BMI > 35 to fully exclude the influence of an increase in obesity-related inflammatory factors.

### 4.2. Biochemical Analyses

Venous blood samples were collected from each patient into a tube with clot activator (S-Monovette, SARSTEDT, Numbrecht, Germany), centrifuged to obtain serum samples, and stored at −80 °C until assayed. The tested chemokines were measured with a multiplexing method (Luminex Human Discovery Assay (3-Plex), R&D Systems, Abingdon, UK). The CCR3 receptor was measured with the enzyme-linked immunosorbent assay (ELISA) (Aviva Systems Biology Corp., San Diego, CA, USA). Serum levels of classical tumor markers were measured with chemiluminescent microparticle immunoassay (CMIA) (Abbott, Chicago, IL, USA), and for the analysis of CRP concentration, immunoturbidimetric method (Abbott, Chicago, IL, USA) was used according to the manufacturer’s protocols. In Luminex and ELISA, according to the manufacturer’s protocols, duplicate samples were assessed for each standard, control, and sample.

### 4.3. Statistical Analysis

Statistical analysis was performed by RStudio (Boston, MA, USA). The preliminary statistical analysis (using the Shapiro–Wilk test) revealed that the tested parameters and tumor marker levels did not follow normal distribution. Consequently, statistical analysis between the groups was performed by using the U-Mann–Whitney test, the Kruskal–Wallis test, and a multivariate analysis of various data by the post-hoc Dwass–Steele–Crichlow–Flinger test. The data were presented as a median and a range. Diagnostic sensitivity (SE), specificity (SP), and the predictive values of positive and negative test results (PPV and NPV, respectively) were calculated by using the cut-off values which were calculated by the Youden’s index (as a criterion for selecting the optimum cut-off point) from the control group, and for each of the tested parameters were as follows: CCL11—12.75 pg/mL, CCL24—1401.83 pg/mL, CCL26—29.15 pg/mL, CCR3—0.17 ng/mL, CA 19-9—5.44 U/mL, CEA—1.11 ng/mL, CRP—2.5 mg/L. We also defined the receiver-operating characteristics (ROC) curve for all the tested parameters, tumor markers, and CRP to evaluate the diagnostic accuracy. Statistically significant differences were defined as comparisons resulting in *p* < 0.05.

## 5. Conclusions

According to our knowledge, the present study is the first that compares the diagnostic characteristics of all eotaxins and their receptor with the well-established tumor markers (CEA and CA 19-9) and the marker of inflammation (CRP) in CRC patients. In addition, due to a limited number of papers, it is extremely difficult to determine the direction in which changes in their concentrations are progressing in the course of colorectal cancer, especially if the available literature is limited to the concentrations of only one of the tested parameters. It is certain that further studies on the concentrations of eotaxins in the course of CRC are necessary to confirm and clarify their diagnostic usefulness and clinical application as potential tumor markers of CRC. However, the most promising factor seems to be CCR3, especially in combined use with CRP or CEA.

## Figures and Tables

**Table 1:** Serum levels of tested parameters in cancer and control groups.

Parameter		Colorectal Cancer	Control Group	*p* *
CCL11 (pg/mL)	Median Min–Max	12.95 5.97–39.30	13.74 5.68–44.42	0.346
CCL24 (pg/mL)	Median Min–Max	1476.36 193.97–4102.92	1341.60 222.75–4189.99	0.458
CCL26 (pg/mL)	Median Min–Max	22.00 6.47–31.92	22.00 10.95–78.18	0.380
CCR3 (ng/mL)	Median Min–Max	0.22 0.10–0.53	0.12 0.10–1.19	**0.012**
CA 19-9 (U/mL)	Median Min–Max	4.14 2.00–8199.90	4.94 2.00–16.81	0.493
CEA (ng/mL)	Median Min–Max	1.90 0.50–1176.50	0.83 0.50–7.82	**<0.001**
CRP (mg/L)	Median Min–Max	5.40 1.00–103.50	1.31 0.20–5.80	**<0.001**

* U Mann–Whitney test.

**Table 2:** Diagnostic criteria of tested parameters in patients with colorectal cancer.

Tested Parameters	Diagnostic Criteria (%)	Colorectal Cancer
CCL11	SE	53.19
	SP	45.83
	PPV	65.79
	NPV	33.33
CCL24	SE	58.70
	SP	58.33
	PPV	72.97
	NPV	42.42
CCL26	SE	4.26
	SP	92.31
	PPV	50.00
	NPV	34.78
CCR3	SE	68.00
	SP	62.07
	PPV	75.56
	NPV	52.94
CA 19-9	SE	40.00
	SP	58.62
	PPV	62.50
	NPV	36.17
CEA	SE	80.00
	SP	65.52
	PPV	80.00
	NPV	65.52
CRP	SE	72.00
	SP	79.31
	PPV	85.71
	NPV	62.16
CCR3+CEA	SE	92.00
	SP	44.83
	PPV	74.19
	NPV	76.47
CCR3+CRP	SE	94.00
	SP	48.28
	PPV	75.81
	NPV	82.35

SE—sensitivity; SP—specificity; PPV—positive predictive value; NPV—negative predictive value.

**Table 3:** AUC of tested parameters in patients with colorectal cancer.

Tested Parameters	ROC Criteria in Total Colorectal Cancer Group			
	AUC	SE	95% C.I. (AUC)	*p* (AUC = 0.5)
CCL11	0.430	0.073	0.283–0.5787	0.173
CCL24	0.555	0.070	0.4086–0.701	0.228
CCL26	0.441	0.072	0.3081–0.5744	0.813
CCR3	0.683	0.060	0.5646–0.803	**0.007**
CA 19-9	0.546	0.066	0.4123–0.6808	0.756
CEA	0.758	0.054	0.6487–0.8665	**<0.001**
CRP	0.830	0.045	0.7425–0.9175	**<0.001**
CCR3+CEA	0.779	0.057	0.6772–0.8814	**<0.001**
CCR3+CRP	0.846	0.049	0.7631–0.9294	**<0.001**

*p*—statistically significantly larger AUC’s compared to AUC = 0.5. AUC—area under curve; SE—standard error; C.I.—confidence interval.

**Table 4:** Characteristics of colorectal cancer and healthy patient groups.

Study Group		No. of Patients
Colorectal Cancer		50
	Gender:	
	Female	18
	Male	32
	Type:	
	Colon Cancer	16
	Rectal Cancer	32
	Sigmoid Cancer	2
	TNM Stage:	
	0	1
	I	15
	II	13
	III	19
	IV	2
	Depth of tumor invasion:	
	In situ	1
	T1	2
	T2	19
	T3	24
	T4	4
	Nodal involvement:	
	N0	33
	N1	10
	N2	7
	Distant metastasis:	
	M0	48
	M1	2
	Age:	33–79
Control Group		30
	Gender:	
	Female	8
	Male	22
	Age:	34–80

TNM—Tumor Node Metastasis classification.

## Data Availability

The data presented in this study are available on request from the corresponding author. Key data are stated in the text.

## Supplementary Materials

The following are available online at https://www.mdpi.com/article/10.3390/jcm10122675/s1, Table S1: Serum levels of tested parameters in colon cancer subtype and control, Table S2: Serum levels of tested parameters in rectal cancer subtype and control, Table S3: Serum levels of tested parameters in cancer subtype groups, Table S4: Serum levels of tested parameters in Early TNM and control groups, Table S5: Serum levels of tested parameters in Advanced TNM and control groups, Table S6: Serum levels of tested parameters in different TNM stages of CRC.
