# Supplementary material for: Eotaxins and Their Receptor as Biomarkers of Colorectal Cancer
Source: J Clin Med. 2021 Jun 17;10(12):2675. doi: 10.3390/jcm10122675 (PMC8235018; doi:10.3390/jcm10122675)
Supplement: Supplementary file 1 [file jcm-10-02675-s001.zip › jcm-1199949-supplementary.pdf]

Supplementary Table S1. Serum levels of tested parameters in colon cancer subtype and control group.

Parameter		Colon Cancer	Control Group	p*
CCL11 [pg/mL]	Median<br>Min - Max	16.62<br>7.94 - 39.30	13.74<br>5.68 – 44.42	0.125
CCL24 [pg/mL]	Median<br>Min – Max	1522.83<br>382.65 - 3357.22	1341.60<br>222.75 – 4189.99	0.436
CCL26 [pg/mL]	Median<br>Min – Max	22.00<br>6.47 - 31.92	22.00<br>10.95 – 78.18	0.654
CCR3 [ng/mL]	Median<br>Min – Max	0.22<br>0.10 - 0.53	0.12<br>0.10 – 1.19	0.057
CA 19-9 [U/mL]	Median<br>Min – Max	3.65<br>2.00 - 8199.90	4.94<br>2.00 - 16.81	0.402
CEA [ng/mL]	Median<br>Min – Max	2.70<br>0.50 - 1176.50	0.83<br>0.50 - 7.82	**<0.001**
CRP [mg/L]	Median<br>Min - Max	5.60<br>1.20-65.20	1.31<br>0.20 - 5.80	**<0.001**

** U Mann-Whitney test*

Supplementary Table S2. Serum levels of tested parameters in rectal cancer subtype and control group.

Parameter		Rectal Cancer	Control Group	p*
CCL11 [pg/mL]	Median<br>Min - Max	12.03<br>5.97 - 29.45	13.74<br>5.68 – 44.42	0.715
CCL24 [pg/mL]	Median<br>Min – Max	1461.22<br>193.97 - 4102.92	1341.60<br>222.75 – 4189.99	0.589
CCL26 [pg/mL]	Median<br>Min – Max	22.00<br>8.55 - 29.96	22.00<br>10.95 – 78.18	0.342
CCR3 [ng/mL]	Median<br>Min – Max	0.22<br>0.12 - 0.43	0.12<br>0.10 – 1.19	**0.012**
CA 19-9 [U/mL]	Median<br>Min – Max	4.72<br>2.00 - 520.80	4.94<br>2.00 - 16.81	0.679
CEA [ng/mL]	Median<br>Min – Max	1.83<br>0.50 - 72.30	0.83<br>0.50 - 7.82	**0.003**
CRP [mg/L]	Median<br>Min - Max	5.40<br>1.00 - 103.50	1.31<br>0.20 - 5.80	**<0.001**

** U Mann-Whitney test*

Supplementary Table S3. Serum levels of tested parameters in cancer subtype groups.

Parameter		Colon Cancer	Rectal Cancer	p*
CCL11	Median	16.62	12.03	0.110
[pg/mL]	Min - Max	7.94 - 39.30	5.97 - 29.45	
CCL24	Median	1522.83	1461.22	0.620
[pg/mL]	Min – Max	382.65 - 3357.22	193.97 - 4102.92	
CCL26	Median	22.00	22.00	0.780
[pg/mL]	Min – Max	6.47 - 31.92	8.55 - 29.96	
CCR3	Median	0.22	0.22	0.490
[ng/mL]	Min – Max	0.10 - 0.53	0.12 - 0.43	
CA 19-9	Median	3.65	4.72	0.630
[U/mL]	Min – Max	2.00 - 8199.90	2.00 - 520.80	
CEA	Median	2.70	1.83	0.310
[ng/mL]	Min – Max	0.50 - 1176.50	0.50 - 72.30	
CRP	Median	5.60	5.40	0.790
[mg/L]	Min - Max	1.20-65.20	1.00 - 103.50	

** U Mann-Whitney test*

Supplementary Table S4. Serum levels of tested parameters in Early TNM and control groups.

Parameter		Early TNM	Control Group	p*
CCL11	Median	12.03	13.74	0.445
[pg/mL]	Min - Max	7.94 - 29.80	5.68 – 44.42	
CCL24	Median	1417.57	1341.60	0.907
[pg/mL]	Min – Max	383.00 - 3204.00	222.75 – 4189.99	
CCL26	Median	22.00	22.00	0.431
[pg/mL]	Min – Max	8.55 - 31.90	10.95 – 78.18	
CCR3	Median	0.22	0.12	0.054
[ng/mL]	Min – Max	0.10 - 0.53	0.10 – 1.19	
CA 19-9	Median	3.39	4.94	0.142
[U/mL]	Min – Max	2.00 - 34.80	2.00 - 16.81	
CEA	Median	1.84	0.83	**0.006**
[ng/mL]	Min – Max	0.50 - 31.10	0.50 - 7.82	
CRP	Median	5.90	1.31	**<0.001**
[mg/L]	Min - Max	1.00 - 104.00	0.20 - 5.80	

** U Mann-Whitney test*

Supplementary Table S5. Serum levels of tested parameters in Advanced TNM and control groups.

Parameter		Advanced TNM	Control Group	p*
CCL11	Median	13.80	13.74	0.570
[pg/mL]	Min - Max	5.97 - 39.30	5.68 – 44.42	
CCL24	Median	1618.36	1341.60	0.278
[pg/mL]	Min – Max	194.00 - 4103.00	222.75 – 4189.99	
CCL26	Median	22.00	22.00	0.676
[pg/mL]	Min – Max	6.47 - 28.90	10.95 – 78.18	
CCR3	Median	0.22	0.12	**0.024**
[ng/mL]	Min – Max	0.12 - 0.25	0.10 – 1.19	
CA 19-9	Median	5.79	4.94	0.135
[U/mL]	Min – Max	2.00 - 8200.00	2.00 - 16.81	
CEA	Median	2.70	0.83	**0.002**
[ng/mL]	Min – Max	0.50 - 1177.00	0.50 - 7.82	
CRP	Median	3.20	1.31	**0.001**
[mg/L]	Min - Max	1.00 - 83.60	0.20 - 5.80	

** U Mann-Whitney test*

Supplementary Table S6. Serum levels of tested parameters in different TNM stages of CRC.

Parameter		Early TNM	Advanced TNM	p*
CCL11	Median	12.03	13.80	0.530
[pg/mL]	Min - Max	7.94 - 29.80	5.97 - 39.30	
CCL24	Median	1417.57	1618.36	0.130
[pg/mL]	Min – Max	383.00 - 3204.00	194.00 - 4103.00	
CCL26	Median	22.00	22.00	0.950
[pg/mL]	Min – Max	8.55 - 31.90	6.47 - 28.90	
CCR3	Median	0.22	0.22	0.650
[ng/mL]	Min – Max	0.10 - 0.53	0.12 - 0.25	
CA 19-9	Median	3.39	5.79	0.060
[U/mL]	Min – Max	2.00 - 34.80	2.00 - 8200.00	
CEA	Median	1.84	2.70	0.370
[ng/mL]	Min – Max	0.50 - 31.10	0.50 - 1177.00	
CRP	Median	5.90	3.20	0.500
[mg/L]	Min - Max	1.00 - 104.00	1.00 - 83.60	

** U Mann-Whitney test*
